# Sex correction improves the accuracy of clinical dopamine transporter imaging

**DOI:** 10.1186/s13550-021-00825-3

**Published:** 2021-08-23

**Authors:** Emma A. Honkanen, Tommi Noponen, Risto Hirvilammi, Kari Lindholm, Riitta Parkkola, Juho Joutsa, Andrea Varrone, Valtteri Kaasinen

**Affiliations:** 1 Clinical Neurosciences, University of Turku, Turku, Finland; 2 Neurocenter, Turku University Hospital, Turku, Finland; 3 Department of Neurology, Satasairaala Central Hospital, Pori, Finland; 4 Turku PET Centre , Turku University Hospital, Turku, Finland; 5 Department of Clinical Physiology and Nuclear Medicine, University of Turku and Turku University Hospital, Turku, Finland; 6 Department of Medical Physics, Turku University Hospital, Turku, Finland; 7 Department of Radiology, University of Turku and Turku University Hospital, Turku, Finland; 8 Turku Brain and Mind Center, University of Turku, Turku, Finland; 9 Department of Clinical Neuroscience, Centre for Psychiatry Research, Karolinska Institutet and Stockholm Health Care Services, Stockholm, Sweden

**Keywords:** Dopamine transporter, SPECT, Healthy, Sex correction

## Abstract

**Background:**

In clinical diagnostic imaging, dopamine transporter (DAT) SPECT scans are commonly evaluated using automated semiquantitative analysis software. Age correction is routinely implemented, but usually no sex correction of DAT binding is performed. Since there are sex differences in presynaptic dopaminergic function, we investigated the effect of DAT sex correction in a sample of healthy volunteers who underwent brain [^123^I]-FP-CIT SPECT.

**Methods:**

Forty healthy elderly individuals (21 men and 19 women) underwent brain [^123^I]-FP-CIT SPECT, and each subject was examined clinically for motor and non-motor parkinsonian symptoms and signs. Regional specific DAT binding ratios (SBR = [ROI-occ]/occ) were calculated using age correction, and the results were compared to those in normal databases with and without sex correction. The level of regional abnormality was set at 2 standard deviations below the mean values of the reference databases.

**Results:**

In the analysis without sex correction, compared to the mean ratio of the reference database, ten healthy individuals (8 men and 2 women) had abnormally low DAT binding ratios, and four individuals (3 men and 1 woman) had borderline low DAT binding ratios in at least one striatal region. When sex correction was implemented, the ratio of one individual was abnormal, and the ratio of one individual was borderline (both males). There were no clinically significant differences in motor or non-motor symptoms between healthy volunteers with abnormal and normal binding.

**Conclusions:**

A considerable number of elderly healthy male subjects can be interpreted to be dopaminergically abnormal if no sex correction of DAT binding is performed. Sex differences in striatal dopaminergic function should be taken into account when DAT imaging is used to assist clinical diagnostics in patients with suspected neurological disorders.

**Supplementary Information:**

The online version contains supplementary material available at 10.1186/s13550-021-00825-3.

## Background

Brain dopamine transporter (DAT) imaging has a significant role in the diagnostic process of parkinsonian patients, as it strongly directs the clinical diagnosis [1]. This means that a patient with an abnormal finding on DAT imaging is likely to receive a diagnosis of a degenerative parkinsonian disorder. In clinical diagnostic practice, DAT imaging with [^123^I]-FP-CIT SPECT is usually interpreted visually together with a region-of-interest-based semiquantitative method. Common neuroimaging software used in the semiautomated DAT SPECT analysis includes BRASS from Hermes Medical Solutions (Stockholm, Sweden) and DaTQUANT from GE Healthcare (Tirat Hacarmel, Israel). These programs calculate specific binding ratios (SBRs) and compare them against reference values derived from healthy control databases, after which regional abnormal values are flagged to assist clinical diagnosis.

There are studies that suggest that striatal DAT availability is lower in men than in women [2–7], although the evidence is not consistent [8–11]. Age correction is implemented in both BRASS and DaTQUANT, but sex correction is not, most likely because the effect of sex on DAT binding has been considered minor for clinical diagnostic imaging. Indeed, there are recent data that suggest that age and sex correction are not essential for DAT SPECT since the variance caused by age and sex is small in comparison with the effect of disease (> 50% reduction in DAT binding in patients with Parkinson’s disease (PD) compared to healthy individuals) [12]. Although this may be the case for the difference between normal dopamine function and clear degeneration of the dopamine system, it is possible that sex differences in dopaminergic function influence the interpretation of findings in healthy subjects, leading to false-positive findings and incorrect diagnoses in the clinical setting. Therefore, in the present study, we aimed to investigate whether sex correction improves the interpretation of findings in healthy individuals.

## Methods

### Patients

Forty healthy individuals (21 males and 19 females) were recruited as volunteer control subjects for a clinical DAT imaging trial, and the sample size was designed for that study. The inclusion criteria were an age of 50–85 years, no use of medications affecting the central nervous system, and no neurological symptoms or relevant prior neurological or psychiatric diseases. All participants underwent [^123^I]-FP-CIT SPECT and brain MRI on the same day. SPECT scanning was performed with a Siemens Symbia T6 SPECT/TT system (Siemens Healthineers, Erlangen, Germany). Subjects received an intravenous injection of 185 MBq of [^123^I]FP-CIT. SPECT imaging started 3 h after the injection. MRI data were acquired with a Siemens 3 T Skyra Fit scanner (Siemens Medical Imaging, Erlangen, Germany), and the imaging protocol included three-dimensional T1, T2, and FLAIR images. There were no clinically significant findings on brain MRI except for a small aneurysm in the internal carotid artery in one subject, small parasagittal cavernoma in one subject and small cortical meningiomas in two subjects. None of these findings were in the striatal area and were considered non-significant with respect to DAT imaging. As a positive family history of PD is a known risk factor for PD [13], in a sub-analysis, subjects with positive family history (*n* = 6) were excluded.

All subjects were clinically examined 2–4 h before DAT scanning. The investigation included a clinical interview, the Unified Parkinson’s Disease Rating Scale (MDS-UPDRS) Part III, Mini-Mental State Examination (MMSE), Beck Depression Inventory (BDI), Beck Anxiety Inventory (BAI), single-question screen for REM sleep behavior disorder (RBD) [14], and Non-Motor Symptoms Scale (NMSS) [15]. All participants gave written informed consent for participation in the study. The study was approved by the local Ethics Committee and was conducted according to the Declaration of Helsinki.

### Image analysis

First, normal clinical analysis was performed using visual and an age-corrected semiquantitative method. Then, DAT binding was compared between men and women separately using the lower level of the 95% confidence interval of the SBR in relation to age and sex [2].

SPECT images were reconstructed with Hermes Hybrid Recon Neurology (version 2.1.1, Hermes Medical Solutions, Stockholm, Sweden) software with 15 iterations, 5 subsets, Gaussian post-filter with 7-mm full width at half maximum, Chang attenuation, resolution recovery and Monte Carlo-based scatter correction. Image analyses were performed using BRASS automated analysis software (version 2.6, Hermes Medical Solutions, Stockholm, Sweden). The reconstruction protocol was set to be compatible with the reference database used in BRASS. The SPECT device was calibrated using instructions for camera corrections from the ENC-DAT database utilizing striatal phantom and well counter measurements [16, 17]. The ENC-DAT database includes the striatal DAT binding values of 139 healthy individuals (74 males and 65 females; ratio 1.1) from different sites determined using [123I]FP-CIT SPECT [2]. Camera calibration produced a correction factor that normalized the specific binding ratios (SBRs) to those acquired with the average SPECT device used in the ENC-DAT project. SBRs for four regions were calculated (the right and left caudate, and right and left putamen) using the occipital cortex as the reference region: SBR = (VOI_caudate or putamen_ − VOI_occipital_)/VOI_occipital_ [2]. DAT binding was considered abnormal if the SBR was below 2 SDs of the reference database values in any of the four regions. This is also the level of abnormality that is used in BRASS in routine clinical practice. Putamen DAT binding asymmetry indices (with following formula: (right–left putamen)/(right + left putamen) [18]) and putamen/caudate ratios were calculated.

SPECT images were re-reconstructed using parameters compatible with the original ENC-DAT database, i.e., 10 iterations, 10 subsets, Butterworth post-processing filter with a cutoff of 0.50 cm^−1^ and order 10 and using the same corrections as for BRASS reconstructions. The lower 95% confidence interval of the SBR in relation to age was calculated for men and women separately using equations derived from the ENC-DAT data [2].

To illustrate the magnitude of the sex differences in each voxel within the striatum, we computed voxelwise age-corrected sex-difference maps using Statistical Parametric Mapping software (SPM12, https://www.fil.ion.ucl.ac.uk/spm/software/spm12/). An average image of the reconstructed scans was calculated and used to compute a nonlinear transformation to the Montreal Neurological Institute (MNI) standard space by using an in-house [^123^I]FP-CIT-SPECT template [19]. This transformation was then applied to all individual reconstructed images. The resulting normalization was inspected visually. The occipital cortex was used as the reference tissue to calculate voxel-to-occipital ratio (SBR + 1) images [20]. An 8-mm Gaussian smoothing kernel was applied to improve the signal-to-noise ratio. A general linear model was created to estimate the magnitude of the sex difference (women > men), controlling for age. The resulting image was then divided by the average voxel-to-occipital ratio image of the men and masked [21], resulting in a voxelwise map of relative striatal sex differences.

### Statistical analyses

SPSS Statistics (IBM version 26, SPSS Inc., Chicago, IL, USA) was used for all statistical analyses. The assumption of normality was tested with Shapiro–Wilk tests together with histograms. The differences between the groups were calculated using independent-samples *t*-test, Mann–Whitney *U* test, Chi-square test and Fisher’s exact test as appropriate. *P* values were corrected for multiple comparisons by applying a Bonferroni correction for two regions (caudate and putamen) in DAT binding and for eight separate symptom scales and questionnaires. Level of statistical significance was set at *P* value less than 0.05.

## Results

The demographic and clinical characteristics of the studied subjects are presented in Table 1.

**Table 1:** Demographic and clinical characteristics of healthy volunteers with normal and abnormal striatal DAT binding

Variable group	Variable	All	Normal DAT	Abnormal or borderline DAT	*P* value^a^	*P*-value corrected^b^
Demographics	*n*	40	26	14	–	–
	Age (years)	66.8 (9.0)	68.2 (9.7)	64.2 (7.3)	0.18	–
	Sex (m/f)	21/19	10/16	11/3	0.02	–
Motor symptoms	MDS-UPDRS motor score	6.6 (5.5)	7.2 (5.7)	5.5 (5.1)	0.23	1.00
Premotor and non-motor symptoms	NMSS total score	16.2 (16.5)	19.1 (19.3)	10.9 (7.3)	0.44	1.00
	RBD (yes/no)	5/35	3/23	2/12	1.00	1.00
	Constipation	0.5 (1.5)	0.6 (1.7)	0.4 (1.1)	0.94	1.00
	Hyposmia	0.3 (1.4)	0.3 (1.6)	0.3 (1.1)	0.99	1.00
Cognition	MMSE	28.0 (2.1)	28.2 (2.2)	27.6 (1.8)	0.22	1.00
Mood and anxiety	BDI	2.6 (3.9)	3.8 (4.4)	0.5 (0.9)	0.02	0.17
	BAI	4.2 (4.4)	5.6 (4.4)	1.6 (3.1)	< 0.001	< 0.001
DAT binding	Caudate	2.58 (0.32)	2.73 (0.25)	2.29 (0.21)	< 0.001	< 0.001
	Putamen	2.36 (0.32)	2.54 (0.24)	2.04 (0.16)	< 0.001	< 0.001
	Putamen asymmetry index	0.01 (0.04)	-0.0008 (0.04)	0.03 (0.04)	0.02	–
	Putamen/caudate ratio	0.92 (0.05)	0.93 (0.05)	0.89 (0.05)	0.03	–

Values are means (SD) or *n*

^a^Mann-Whitney *U*-test, independent-samples *t*-test or Fisher’s exact test

^b^Bonferroni correction to adjust for multiple comparisons for two DAT binding regions and for eight symptom scales and questionnaires. Numbers of missing values: BAI = 1

**Fig. 1:**
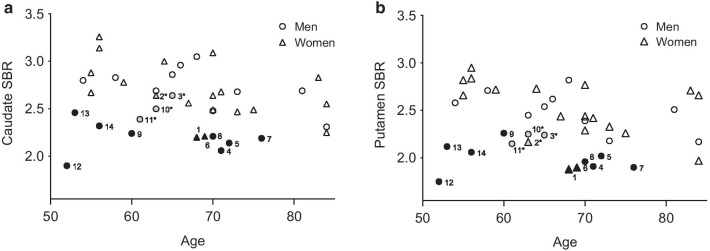
Specific binding ratios (SBRs) in **A** caudate nucleus and **B** putamen in healthy women and men with abnormal DAT binding (men = solid circle, women = solid triangle), with borderline DAT binding (men = grey circle, women = grey triangle) and with normal DAT binding (men = open circle, women = open triangle). Corresponding subject numbers with abnormal and borderline (*) DAT binding are presented in Table 2

When SBRs were compared to those in the ENC-DAT-based BRASS database, which includes both men and women, 10 subjects in our sample were indicated to have abnormally low DAT binding in at least one striatal region (*z*-values below the level of 2 SDs). In addition, four subjects had borderline values in at least one region (*z* = − 1.87 to − 1.98). Detailed data for these 14 subjects are shown in Table 2. In only one subject (subject 9 in Table 2), the abnormal binding was limited to the caudate nucleus. Individuals with normal DAT binding had a 19.2% higher SBR in the caudate and 24.1% higher SBR in putamen than those with binding values that were flagged as abnormal or borderline (*P* < 0.001) (Fig. 1, Table 1). When values from right and left sides were analyzed separately, subjects with normal DAT binding had 18.5% higher SBR in the right caudate, 20.4% higher SBR in left caudate, 20.5% higher SBR in right putamen and 27.8% higher SBR in left putamen compared to subject with abnormal or borderline DAT binding (all corrected *P* < 0.001).

**Table 2:** Individual demographic and clinical characteristics of healthy subjects with abnormal or borderline DAT binding

Subject number	Age	Sex (male/female)	MDS-UPDRS part III score	MMSE	BDI	BAI	NMSS	Constipation	Hyposmia	RBD (yes/no)	Right cau SBR	Left cau SBR	Mean cau SBR	Right put SBR	Left put SBR	Mean put SBR	Put asymmetry index	Put/cau ratio
1	68	F	12	24	0	0	16	0	0	No	2.19	2.20	2.20	1.89	1.87	1.88	0.01	0.86
2^b^	63	F	1	29	2	11	14	1	0	No	2.42	2.85	2.64	2.28	2.05	2.17	0.05	0.82
3^b^	65	M	2	27	0	0	4	0	0	Yes	2.72	2.55	2.64	2.43	2.05	2.24	0.08	0.85
4	71	M	1	29	0	0	5	0	4	No	2.07	2.05	2.06	1.95	1.86	1.91	0.02	0.92
5	72	M	2	28	0	1	24	0	0	Yes	2.21	2.06	2.14	2.18	1.86	2.02	0.08	0.95
6	69	F	4	27	2	4	25	4	0	No	2.24	2.18	2.21	1.93	1.87	1.90	0.02	0.86
7	76	M	13	30	0	0	1	0	0	No	2.27	2.10	2.19	1.99	1.80	1.90	0.05	0.87
8	70	M	1	25	2	4	6	0	0	No	2.17	2.24	2.21	2.05	1.88	1.96	0.04	0.89
9	60	M	8	30	0	0	16	0	0	No	2.28	2.19	2.24	2.20	2.32	2.26	− 0.03	1.01
10^b^	63	M	6	26	1	0	11	0	0	No	2.48	2.52	2.50	2.44	2.05	2.25	0.09	0.90
11^b^	61	M	2	29	0	1	6	0	0	No	2.33	2.44	2.39	2.14	2.16	2.15	0.00	0.90
12^a^	52	M	16	28	0	1	7	0	0	No	1.93	1.88	1.90	1.80	1.70	1.75	0.03	0.92
13	53	M	8	27	0	0	9	0	0	No	2.35	2.56	2.46	2.06	2.17	2.12	− 0.03	0.86
14^a^	56	M	1	28	0	0	8	0	0	No	2.18	2.45	2.32	2.10	2.02	2.06	0.02	0.89
Mean (SD)	64.2 (7.3)	11/3	5.5 (5.1)	27.6 (1.8)	0.5 (0.9)	1.6 (3.1)	10.9 (7.3)	0.4 (1.1)	0.3 (1.1)	2/12	2.27 (0.19)	2.31 (0.26)	2.29 (0.21)	2.10 (0.19)	1.98 (0.17)	2.04 (0.16)	0.03 (0.04)	0.89 (0.05)

Cau, caudate; put, putamen

^a^Remained abnormal after sex correction

^b^Borderline DAT binding before sex correction

**Fig. 2:**
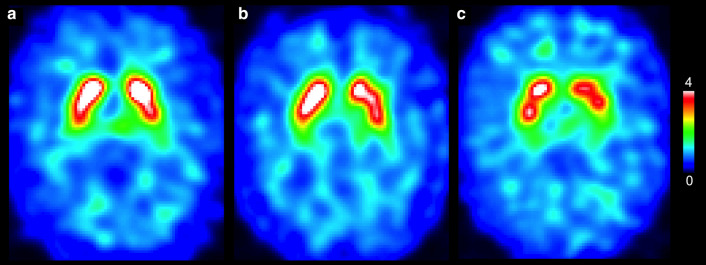
Three representative striatal level [^123^I]FP-CIT SPECT images from three subjects. **A** Image evaluated visually as normal, but semiquantitatively abnormal (Subject 8 in Table 2, male). **B** Image evaluated visually as borderline, but semiquantitatively abnormal (Subject 6, female). **C** Image evaluated visually and semiquantitatively abnormal (Subject 12, male)

The majority of the subjects with abnormal scans were male (11 males vs 3 females, *P* = 0.02), but there were no differences in PD-related motor or non-motor symptoms or clinically significant differences in cognitive, mood or anxiety scores (Table 1). Putamen asymmetry indices were higher in subjects with abnormal or borderline DAT (Table 1). Also, putamen/caudate ratios were lower in subjects with abnormal or borderline DAT binding (Table 1). Seven subjects out 14 with abnormal or borderline DAT binding values were visually evaluated abnormal or borderline (Fig. 2, Additional file 1: Table S1) There were no differences between men and women in demographics or clinical characteristics (Table 3).

**Table 3:** Demographic and clinical characteristics of women and men separately

Variable group	Variable	All	Women	Men	*P* value^a^	*P*-value corrected^b^
Demographics	*n*	40	19	21	–	–
	Age (years)	66.8 (9.0)	68.0 (9.4)	65.8 (8.8)	0.44	–
Motor symptoms	MDS-UPDRS motor score	6.6 (5.5)	6.4 (4.8)	6.9 (6.3)	0.92	–
Premotor and non-motor symptoms	NMSS total score	16.2 (16.5)	13.7 (11.7)	18.4 (19.9)	0.81	1.00
	RBD (yes/no)	5/35	1/18	4/17	0.35	1.00
	Constipation	0.5 (1.5)	0.9 (2.1)	0.1 (0.4)	0.25	1.00
	Hyposmia	0.3 (1.4)	0.5 (1.8)	0.2 (0.9)	0.77	1.00
Cognition	MMSE	28.0 (2.1)	27.9 (2.3)	28.0 (1.9)	0.96	1.00
Mood and anxiety	BDI	2.6 (3.9)	3.2 (4.1)	2.1 (3.7)	0.23	1.00
	BAI	4.2 (4.4)	5.1 (4.1)	3.4 (4.5)	0.13	1.00
DAT binding	Caudate	2.58 (0.32)	2.67 (0.30)	2.49 (0.31)	0.07	0.14
	Putamen	2.36 (0.32)	2.47 (0.33)	2.26 (0.29)	0.04	0.08
	Putamen asymmetry index	0.01 (0.04)	0.003 (0.03)	0.02 (0.05)	0.25	–
	Putamen/caudate ratio	0.92 (0.05)	0.92 (0.06)	0.91 (0.04)	0.42	–

Values are means (SD) or *n*

^a^Mann-Whitney *U*-test, independent-samples *t*-test or Fisher’s exact test

^b^Bonferroni correction to adjust for multiple comparisons for two DAT binding regions and for eight symptom scales and questionnaires. Numbers of missing values: BAI = 1

None of the subjects with a positive family history of PD had abnormal DAT binding, but one subject was borderline. When all subjects with a positive family history of PD (*n* = 6) were excluded from the analysis, the results remained the same. Remaining subjects with normal DAT binding (*n* = 21) had 19.0% higher SBR in the caudate (corrected *P* < 0.001) and 22.7% higher SBR in the putamen (corrected *P* < 0.001) compared to the subjects with abnormal/borderline DAT binding (*n* = 13). Similarly to main results, there were no differences in motor or non-motor symptoms (all corrected *P* = 1.00).

When re-reconstructed SBRs were compared to the age- and sex-corrected lower limits of the 95% confidence intervals of the ENC-DAT database, the values of only two healthy individuals remained abnormal. Both of these subjects were male (subjects 12 and 14 in Table 2 and Fig. 1), and the putaminal SBR of subject 14 remained only slightly abnormal (SBR_mean putamen_ = 2.19, lower limit of the 95% CI = 2.20).

**Fig. 3:**
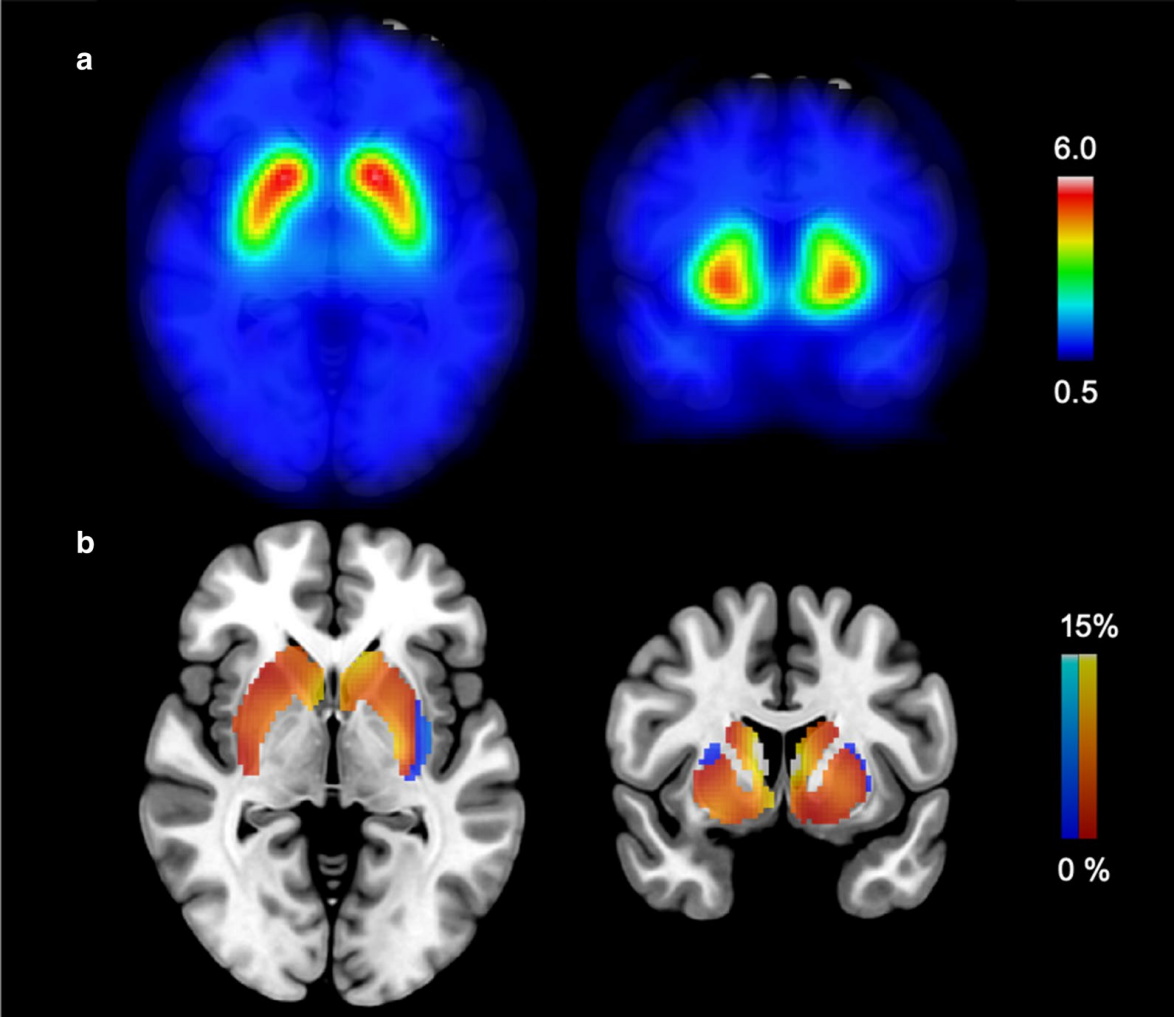
Voxelwise map of sex differences in the striatum. **A** Average SBR + 1 image of the whole sample overlaid on the MNI T1 template. **B** Striatal age-corrected SBR + 1 difference between men and women. Red-yellow scale indicates regions with higher and blue-light scale lower uptake in women compared to men

A striatal age-corrected sex difference was seen across almost the entire striatum (Fig. 3).

## Discussion

The present study shows that the lack of sex correction in clinical DAT imaging increases the risk of false-positive findings for men. The effect could lead to errors in the clinical diagnoses of symptomatic patients with normal dopamine function.

The results of a recent study using the Parkinson’s Progression Markers Initiative (PPMI) material suggested that age and sex correction of the putaminal SBR may not be needed in clinical DAT imaging [12]. On the surface, our results seem to contradict this finding as our results indicate that sex correction is needed. However, it is important to note that the perspectives of the two studies differ considerably. While the earlier study aimed to investigate the usefulness of age and sex correction in the differentiation of PD patients and healthy controls, our study involved only healthy individuals. The earlier study showed that the difference in DAT binding between PD and healthy controls is so large that any possible corrections for age and sex have little role. On the other hand, when subtle differences in dopaminergic function are investigated, such as in our sample of healthy controls, the cutoff between normal and abnormal DAT binding becomes more meaningful. If an individual with symptoms resembling parkinsonism undergoes DAT SPECT and the result erroneously suggests lower than normal binding, this could lead to an inaccurate diagnosis and unnecessary treatments. Additionally, in the PPMI study, the putaminal SBR was found to be 6–14% higher in females than in males [12], but this difference did not have an effect on the difference between PD and healthy subjects. A similar sex difference was also found in the ENC-DAT data, with women showing significantly higher DAT availability than men [2].

Brain dopamine is known to be dysregulated in several neurological and neuropsychiatric disorders that are more common in males, such as PD, attention deficit hyperactivity disorder (ADHD) and autism spectrum disorders. It is possible that sex gonadal hormones along with the Y chromosome regulate dopamine biochemistry and function in the male brain [22–24]. Imaging studies with SPECT and PET have indicated that dopaminergic function is decreased in men compared to women in PD [19, 25–27], and PD symptom severity and treatment response may vary between sexes [28, 29]. Estrogen levels have been considered to be a potential neuroprotective factor together with higher baseline number of dopaminergic neurons in females [23], and also the difference in DAT binding between the sexes seems to maintain, but also to narrow, in aging [2]. It is of importance to note that all women in our study were postmenopausal, and it is thus possible that sex correction is even more relevant in younger age-groups, when estrogen levels potentially affect the dopaminergic function to a greater extent.

In a common clinical protocol, also used by our center, visual reading of SPECT images is performed together with a semiquantitative analysis. In this study, 7 out of 14 subjects with abnormal or borderline semiquantitative results were also visually evaluated as abnormal or borderline. On the other hand, only one out of 26 subjects with normal DAT binding was visually evaluated as abnormal (Additional file 1: Table S1). As visual and semiquantitative analyses are conducted simultaneously together, it is possible that visual analyses are biased by semiquantitative results.

The DAT binding ratio of one male subject (Table 2 subject number 12) remained clearly abnormal even after age and sex corrections were implemented. This individual also had the highest scores on motor examination with the MDS-UPDRS, although he did not report subjective symptoms. We consider it possible that this subject has early prodromal PD. Our results demonstrate that a sex correction in clinical DAT imaging can change the diagnosis from abnormal to normal in some cases, but clearly pathological findings remain pathological irrespective of sex correction or semiquantitative analysis. Nevertheless, an accurate analysis in the borderline of abnormality will assist the clinician to determine whether, for example, a levodopa test or another diagnostic neuroimaging method in necessary.

## Conclusions

The present study shows that the risk of error in DAT imaging of healthy individuals increases without proper sex correction. The results suggest that sex correction should be implemented in automated analysis software that is commonly used to assist clinical diagnosis.

## Supplementary Information

**Additional file 1**. **Table S1.** Visual and semiquantitative results of the subjects. Corresponding numbers of subjects with abnormal DAT binding are presented in Table 2. ^1^remained abnormal after sex correction.

## Data Availability

The datasets generated during and/or analyzed during the current study are available from the corresponding author on reasonable request.

## Abbreviations

ADHD: Attention deficit hyperactivity disorder
BAI: Beck Anxiety Inventory
BDI: Beck Depression Inventory
DAT: Dopamine transporter
MDS-UPDRS: Unified Parkinson’s Disease Rating Scale by Movement Disorder Society
MMSE: Mini-mental state examination
MNI: Montreal Neurological Institute
NMSS: Non-Motor Symptoms Scale
PD: Parkinson’s disease
PPMI: Parkinson’s progression markers initiative
RBD: REM sleep behavior disorder
SBR: Specific binding ratio
SPM: Statistical parametric mapping
