# Supplementary material for: Sex correction improves the accuracy of clinical dopamine transporter imaging
Source: EJNMMI Res. 2021 Aug 23;11:82. doi: 10.1186/s13550-021-00825-3 (PMC8382816; doi:10.1186/s13550-021-00825-3)
Supplement: Supplementary file 1 — Additional file 1. Table S1. Visual and semiquantitative results of the subjects. Corresponding numbers of subjects with abnormal DAT binding are presented in Table 2. 1remained abnormal after sex correction. [file 13550_2021_825_MOESM1_ESM.docx]

**Supplementary Table 1.** Visual and semiquantitative results of the subjects. Corresponding numbers of subjects with abnormal DAT binding are presented in Table 2. ^1^remained abnormal after sex correction.

| Subject | **Visual**  Normal Borderline Abnormal | | | **Semiquantitative**  Normal Borderline Abnormal | | |
| --- | --- | --- | --- | --- | --- | --- |
| 1 |  |  | x |  |  | x |
| 2 |  | x |  |  | x |  |
| 3 | x |  |  |  | x |  |
| 4 | x |  |  |  |  | x |
| 5 |  | x |  |  |  | x |
| 6 |  | x |  |  |  | x |
| 7 |  | x |  |  |  | x |
| 8 | x |  |  |  |  | x |
| 9 | x |  |  |  |  | x |
| 10 | x |  |  |  | x |  |
| 11 | x |  |  |  | x |  |
| 12^1^ |  |  | x |  |  | x |
| 13 | x |  |  |  |  | x |
| 14^1^ |  | x |  |  |  | x |
| 15 | x |  |  | x |  |  |
| 16 | x |  |  | x |  |  |
| 17 | x |  |  | x |  |  |
| 18 | x |  |  | x |  |  |
| 19 | x |  |  | x |  |  |
| 20 | x |  |  | x |  |  |
| 21 | x |  |  | x |  |  |
| 22 | x |  |  | x |  |  |
| 23 | x |  |  | x |  |  |
| 24 | x |  |  | x |  |  |
| 25 | x |  |  | x |  |  |
| 26 | x |  |  | x |  |  |
| 27 | x |  |  | x |  |  |
| 28 | x |  |  | x |  |  |
| 29 | x |  |  | x |  |  |
| 30 | x |  |  | x |  |  |
| 31 | x |  |  | x |  |  |
| 32 | x |  |  | x |  |  |
| 33 | x |  |  | x |  |  |
| 34 |  |  | x | x |  |  |
| 35 | x |  |  | x |  |  |
| 36 | x |  |  | x |  |  |
| 37 | x |  |  | x |  |  |
| 38 | x |  |  | x |  |  |
| 39 | x |  |  | x |  |  |
| 40 | x |  |  | x |  |  |
